# On the performance of seizure prediction machine learning methods across different databases: the sample and alarm-based perspectives

**DOI:** 10.3389/fnins.2024.1417748

**Published:** 2024-07-15

**Authors:** Inês Andrade, César Teixeira, Mauro Pinto

**Affiliations:** University of Coimbra, Centre for Informatics and Systems, Department of Informatics Engineering, Coimbra, Portugal

**Keywords:** seizure prediction, epilepsy, databases, machine learning, EEG

## Abstract

Epilepsy affects 1% of the global population, with approximately one-third of patients resistant to anti-seizure medications (ASMs), posing risks of physical injuries and psychological issues. Seizure prediction algorithms aim to enhance the quality of life for these individuals by providing timely alerts. This study presents a patient-specific seizure prediction algorithm applied to diverse databases (EPILEPSIAE, CHB-MIT, AES, and Epilepsy Ecosystem). The proposed algorithm undergoes a standardized framework, including data preprocessing, feature extraction, training, testing, and postprocessing. Various databases necessitate adaptations in the algorithm, considering differences in data availability and characteristics. The algorithm exhibited variable performance across databases, taking into account sensitivity, FPR/h, specificity, and AUC score. This study distinguishes between sample-based approaches, which often yield better results by disregarding the temporal aspect of seizures, and alarm-based approaches, which aim to simulate real-life conditions but produce less favorable outcomes. Statistical assessment reveals challenges in surpassing chance levels, emphasizing the rarity of seizure events. Comparative analyses with existing studies highlight the complexity of standardized assessments, given diverse methodologies and dataset variations. Rigorous methodologies aiming to simulate real-life conditions produce less favorable outcomes, emphasizing the importance of realistic assumptions and comprehensive, long-term, and systematically structured datasets for future research.

## 1 Introduction

Epilepsy, a prevalent neurological disorder, affects approximately 1% of the global population. Characterized by irregular brain activity, this condition leads to rare and unpredictable epileptic seizures. The primary strategy for managing seizures involves administering anti-seizure medications (ASMs). However, approximately one-third of patients do not respond effectively to this approach, posing significant risks for those with Drug-Resistant Epilepsy (DRE). Apart from the immediate physical dangers like accidental injuries and cerebral damage, epilepsy can trigger psychological and social disorders such as anxiety, depression, and neuropsychological deficits (Perucca et al., 2018; Mehdizadeh et al., 2019).

Improving the quality of life for these patients involves integrating seizure prediction into intervention or alert systems to prevent or minimize the adverse effects of epileptic seizures. The primary goal is to develop an algorithm to predict an impending epileptic seizure and trigger an alert before the seizure onset (Assi et al., 2017). Within this context, the existence of a preictal period is presumed, marked by the transition from normal brain activity to a seizure. Electroencephalogram (EEG) signals can capture this stage, along with the three other stages that define a seizure: ictal (during the seizure), postictal (after the seizure), and interictal (between the postictal and preictal stages of two successive seizures) (Cui et al., 2018). The goal of a prediction algorithm is to identify brain patterns associated with the preictal period. The group by Iasemidis et al. was the first one to show the existence and quantify the duration of a preictal period and, based on this, to develop the first seizure prediction algorithm (Iasemidis, 2003; Iasemidis et al., 2003, 2005; Chaovalitwongse et al., 2005; Sackellares et al., 2006). Assessment of seizure prediction algorithms may divide the preictal period into two periods (that then become algorithms' parameters): the Seizure Prediction Horizon (SPH), a period within which a warning of an upcoming seizure may be issued and intervention may occur, and the Seizure Occurrence Period (SOP), a period following SPH within which the seizure itself may occur (Winterhalder et al., 2003; Schelter et al., 2006).

The selected database profoundly impacts the performance of a seizure prediction algorithm, necessitating the development of a model with universal applicability for direct comparison of outcomes across databases. This study primarily focused on constructing a patient-specific seizure prediction algorithm using a subset of European Epilepsy Database (EPILEPSIAE) data. Subsequently, the algorithm was adapted for application to the Children's Hospital Boston from the Massachusetts Institute of Technology (CHB-MIT), American Epilepsy Society (AES), and Epilepsy Ecosystem databases, enabling the assessment and comparison of their performance.

## 2 Material and methods

**Figure 1:**
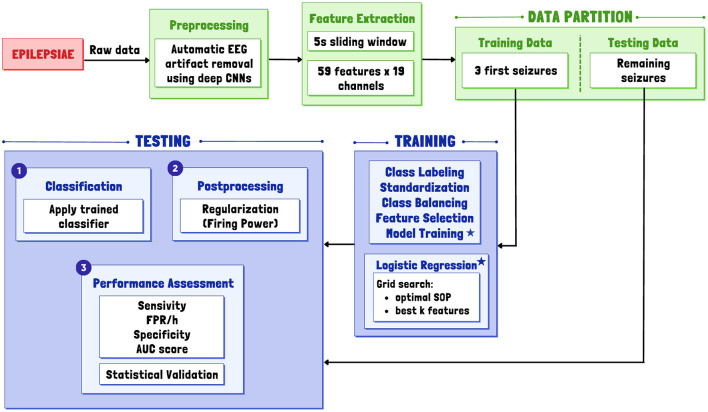
General overview of the proposed patient-specific pipeline for a real-life simulation. Asterisks indicate the inclusion of a Logistic Regression classifier in the model training phase.

We initially developed a patient-specific algorithm for predicting seizures using EPILEPSIAE data and then adapted it for broader applicability to other databases (CHB-MIT, AES, and Epilepsy Ecosystem). To achieve this, we followed a common framework for seizure prediction, illustrated in the Figure 1. This framework included sequential stages: data preprocessing, feature extraction, training, testing, and postprocessing.

Raw EEG data underwent preprocessing and segmentation into non-overlapping 5-second windows to extract relevant features. Subsequently, the data was divided into a training set for parameter optimization and classifier training and a testing set for prediction and classifier evaluation. Variations in algorithms tailored for other databases were influenced by several factors, with how the data is made available being the primary determinant.

This study also involved a phase of statistical assessment to verify that the model's performance is grounded in its ability to recognize patterns associated with seizures rather than random phenomena within EEG signals. This consideration is essential given the rare event nature of seizure prediction, leading to a notable imbalance between interictal and preictal periods.

### 2.1 Data

Detailed information about the data used from each database is available in the Supplementary material.

#### 2.1.1 EPILEPSIAE

In this study, we used data from a subset of 40 patients diagnosed with Temporal Lobe Epilepsy (TLE), comprising 17 females and 23 males, with an average age of 41.4 ± 15.7 years. The EPILEPSIAE dataset (EPILEPSIAE, 2008) comprises scalp EEG recordings acquired from 19 electrodes, aligned with the International 10–20 System, during pre-surgical monitoring sessions conducted at a sampling frequency of 256 Hz.

The selection criteria ensured the inclusion of patients who had experienced a minimum of four independent seizures, with a minimum interval of 4.5 h between each seizure. This approach avoided analyzing seizures belonging to the same seizure cluster.

Approval for the utilization of this data for research purposes was granted by the Ethics Committee of the three hospitals involved in the development of the EPILEPSIAE database (Ethik-Kommission der Albert-Ludwigs-Universität, Freiburg; Comité consultatif sur le traitement de l'information en matiére de recherche dans le domaine de la santé, Hospital Universitário Pitié-Salpětriére; and Ethics Committee of the Centro Hospitalar e Universitário de Coimbra). All studies followed applicable guidelines and regulations, with written informed consent obtained from each patient.

#### 2.1.2 CHB-MIT

From the CHB-MIT dataset (CHB-MIT, 2010), we chose 6 out of the 24 cases. These 6 cases involve data from three female patients, two male patients, and one patient of unknown gender. The data were recorded at a sampling frequency of 256 Hz using 23 or 32 electrodes, following the International 10–20 System.

The selection of these cases followed the criteria applied to the EPILEPSIAE data. Specifically, cases chb12, chb13, and chb18 were excluded, not due to a failure to meet the aforementioned requirements but rather because they exhibited multiple electrode changes or insufficient data. The selected data covers 244 hours, with a total of 32 seizures. The data is available for research purposes, and access is open to all, subject to specified terms.

#### 2.1.3 AES

We also incorporated data from all participants in the AES database (AES, 2014), which includes five dogs and two humans. This dataset consists of Intracranial Electroencephalogram (iEEG) data, with long-term ambulatory recordings collected at a sampling frequency of 400 Hz from five canines with naturally occurring epilepsy and pre-surgical recordings collected at a sampling frequency of 5 kHz from two human subjects. The number of electrodes varied from 15 to 24.

The dataset includes long-term data, but only a portion is accessible, comprising 1-hour recordings divided into 6 individual files categorized as interictal or preictal. This dataset lacks temporal information, making it impractical to establish temporal relationships between files. Therefore, for this study, we utilized 627.6 hours of recordings containing 51 seizures. Access to the data in this dataset is public, and its usage was permitted after the conclusion of the Kaggle competition.

#### 2.1.4 Epilepsy ecosystem

Furthermore, we included data from the Epilepsy Ecosystem (Epilepsy Ecosystem, 2016), corresponding to data from the three patients exhibiting the worst results in the NeuroVista database. Collected at a sampling rate of 400 Hz for 16 channels, these data include iEEG recordings from female patients with an average age of 41 ± 13.5 years.

Much like the AES data, this dataset is structured similarly, with data organized into multiple 10-min files lacking temporal information. However, not all files are open to the public; some are designated as private. Thus, we used a total of 935.3 h of recording. Accessing and using this data involved completing a form, undergoing security procedures, and consenting to the specified terms of use.

### 2.2 Preprocessing

During the preprocessing phase of the EPILEPSIAE data, we employed a methodology based on Convolutional Neural Networks (CNNs) developed by Lopes et al. (2021). This model automatically and efficiently removes artifacts, including eye blinks, eye movements, muscle activity, cardiac activity, and electrode interference, producing results comparable to those achieved by experts.

For the remaining datasets, we implemented low-pass and/or high-pass filters based on the characteristics of each dataset. For iEEG data (AES and Epilepsy Ecosystem), we applied a high-pass filter with a cutoff frequency of 0.5 Hz. For scalp EEG data (CHB-MIT), alongside the high-pass filter, we incorporated a low-pass filter with a cutoff frequency of 60 Hz.

For AES and Epilepsy Ecosystem data, downsampling was necessary due to the higher original sampling frequency exceeding 256 Hz.

### 2.3 Feature extraction

After completing data preprocessing, we partitioned the EEG signals into non-overlapping 5-second windows, allowing for the extraction of relevant features. The selection of this window duration aligns with the contemporary state-of-the-art in the field of seizure prediction (Cook et al., 2013; Teixeira et al., 2014; Direito et al., 2017; Pinto et al., 2022).

Opting for reduced computational complexity and enhanced interpretability, we exclusively extracted univariate linear features. Through a sliding window analysis, we obtained 59 univariate linear features for each channel. The only variation in this procedure among different databases pertains to the total number of features, influenced solely by the varying numbers of channels.

In the frequency domain, the extracted features include the relative spectral power of delta (0.5–4 Hz), theta (4–8 Hz), alpha (8–13 Hz), beta (13–30 Hz), and four gamma subbands: gamma band 1 (30–47 Hz), gamma band 2 (53–75 Hz), gamma band 3 (75–97 Hz), and gamma band 4 (103–128 Hz). Additionally, features include the ratio between these bands, spectral edge frequency, and power at 50%. In the time domain, we computed four statistical moments (mean, variance, skewness, kurtosis), Hjörth parameters (activity, mobility, complexity), and decorrelation time. Regarding time-frequency features, we extracted the energy from five wavelet detail coefficients (from D1 to D5, using the mother wavelet db4) (Lopes et al., 2023; Pinto et al., 2023).

### 2.4 Data splitting

This phase involved partitioning the features into two separate sets for each patient: one designated for training and the other for testing. In contrast to the standardized feature extraction approach, this process demonstrated variations tailored to the distinct attributes of each dataset.

For the EPILEPSIAE and CHB-MIT data, we assigned the initial seizures of each patient to the training set, allocating the subsequent seizures to the testing set. This chronological division was implemented to replicate a realistic seizure prediction scenario, wherein the model first learns from a historical set of seizures before being applied for real-time prediction of future data. For AES and Epilepsy Ecosystem data, the division could not follow the same chronological approach due to the unavailability of temporal seizure data. Instead, the division was made based on the number of available preictal files. We adopted the closest possible ratio of 70/30 for training/testing, ensuring that no preictal files corresponding to the same seizure were present in both sets.

### 2.5 Training

Each patient's training set played a crucial role in determining optimal parameters, including the optimal number of features for all datasets and the SOP duration specifically for the EPILEPSIAE and CHB-MIT data. The identified optimal parameters were then utilized to train the classifier.

In the initial phase, for the EPILEPSIAE and CHB-MIT data, samples were classified into two distinct classes: preictal (1) and interictal (0). The preictal class included the SPH and the SOP. For SOP values, the SPH duration was set at 10 minutes, deemed the most suitable time interval based on the time required for medication to take effect (Boddu and Kumari, 2020; Bouw et al., 2021; Cloyd et al., 2021). For SOP value, we analyzed values between 10 and 55 min at 5-min increments. The preictal period was limited to a maximum of 1 hour, a decision driven by the practicalities of an alert device algorithm. Extending beyond this timeframe could compromise the effectiveness of rescue medication administration and increase patient stress. This procedural step was skipped for the AES and Epilepsy Ecosystem data, as the data had already been categorized and separated into preictal and interictal segments.

Additionally, we applied *z*-score normalization to standardize the training set data. During this training phase, addressing the class imbalance in the EPILEPSIAE and CHB-MIT data required the development of a class balancing strategy. To maintain representativeness and address the disparity between the number of interictal and preictal samples, we implemented class weights calculated inversely to their frequency. For AES and Epilepsy Ecosystem data, the balancing process was simplified and conducted during data splitting by selecting an equal number of preictal and interictal files.

Before classifier training, we employed a grid-search strategy with Leave-One-Out Cross Validation (LOOCV) to identify optimal parameters. This method involves triple cross-validation, incorporating two seizures for training and one for validating the training set, particularly in the case of EPILEPSIAE and CHB-MIT data. In cases where files correspond to interictal and preictal periods rather than seizures, the data were divided as closely as possible to a 70/30 ratio for training and validation.

Identification of the chosen parameters involved evaluating the model's performance, taking into consideration the Equation (1) representing the trade-off between sample sensitivity (SS) and specificity (SP). With the optimal parameters determined, we trained the chosen classifier - a logistic regression.


(1) $$\begin{array}{l} \sqrt{SS_{sample}\cdot SP_{sample}} \end{array}$$

### 2.6 Post-processing

During the testing stage, we employed the same techniques as in the training stage, excluding class balancing. Following the classification phase, we implemented the Firing Power method, proposed by Teixeira et al. (2011), for regularization, aiming to reduce false alarms. This method employs a moving average filter, triggering an alarm when surpassing a predefined threshold, set at 0.7 in our case (Pinto et al., 2023). A refractory period, matching the duration of the preictal period, was also implemented to prevent consecutive and redundant alerts.

### 2.7 Performance evaluation

The final step involved assessing the prediction performance using metrics such as sensitivity, False Prediction Rate per hour (FPR/h), specificity, and Area Under the ROC Curve (AUC) score. Sensitivity and FPR/h are real context metrics corresponding to the alarm-triggering approach. Sensitivity represents the ratio of predicted seizures (true alarms) to the total number of seizures, while FPR/h indicates the number of incorrectly predicted seizures per hour. To enable comparisons with databases lacking temporal seizure information and employing a sample approach, we incorporated specificity and the AUC value.

#### 2.7.1 Alarm and sample-based approaches

We employed two distinct methodologies based on the availability of temporal seizure data. The alarm approach was feasible for the EPILEPSIAE and CHB-MIT datasets due to the comprehensive temporal information available. This allowed us to chronologically assess the data by triggering alarms, enabling the calculation of sensitivity and FPR/h. Conversely, for the AES and Epilepsy Ecosystem datasets, only interictal and preictal files were provided, precluding a temporal-based approach. Therefore, we evaluated these datasets sample-by-sample, determining sensitivity, specificity, and AUC. We also applied this sample-based approach to the EPILEPSIAE and CHB-MIT data to facilitate comparison across all datasets.

#### 2.7.2 Statistical assessment

Additionally, we conducted a statistical assessment to determine whether the algorithm's performance surpassed chance. The surrogate time series analysis method involved 30 random alterations of seizure onset times within the interictal period, establishing the algorithm's superiority over chance if its sensitivity exceeded the statistically significant level (0.05).

## 3 Results and discussion

Table 1 presents the average values of the metrics calculated for all databases. For detailed results of each patient's metrics, refer to the Supplementary material.

**Table 1:** Overall testing results for each dataset.

**Dataset**	***SS*_*Alarm*_**	**FPR/h**	***SS*_*Sample*_**	***SP*_*Sample*_**	**AUC**
EPILEPSIAE	0.13	0.36	0.42	0.69	0.56
CHB-MIT	0.28	0.53	0.45	0.58	0.52
AES	-	-	0.48	0.64	0.56
Epilepsy ecosystem	-	-	0.75	0.37	0.54

Regarding the alarm approach, the sensitivity achieved is notably low for both EPILEPSIAE and CHB-MIT, with a slightly higher value for CHB-MIT. FPR/h values for both datasets exceed the defined ideal threshold of 0.15, considered suitable for practical real-life applications. Unlike sensitivity, the most favorable value is now observed for EPILEPSIAE. However, this better outcome is significantly influenced by instances where, despite the absence of false alarms, no seizures were predicted.

The analysis, limited to patients with at least one predicted seizure, reveals improved sensitivity in both datasets. EPILEPSIAE achieves an average sensitivity of 0.50, while CHB-MIT reaches 0.56. The FPR/h value changes only in the EPILEPSIAE data, reaching 0.49, highlighting the impact of the previously mentioned cases on the results.

Concerning the metrics under the sample approach, there is a marked improvement in sensitivity values for both EPILEPSIAE and CHB-MIT. This finding leads us to the conclusion that addressing this issue through a more realistic approach, where triggering alarms is necessary, results in less favorable outcomes.

The Epilepsy Ecosystem data stands out with a notably high sensitivity value compared to other databases. However, this elevated sensitivity comes at the cost of reduced specificity, in contrast to other databases where specificity is higher. This observed pattern may be attributed to the average number of hours available per seizure, with datasets containing more interictal data contributing to a more accurate classification of the interictal sample. The time per seizure ratios for these datasets were 20.8, 12.31, 7.8, and 4.4 h for EPILEPSIAE, AES, CHB-MIT, and Epilepsy Ecosystem, respectively. Finally, the AUC value consistently exhibited similarity across all databases, reinforcing the trend that when one metric improves, the other tends to decline.

### 3.1 Statistical assessment

Table 2 provides an overview of the number and percentage of patients that successfully passed statistical assessment for each database under the two approaches (alarm and sample). Detailed patient-specific results for this step can be found in the Supplementary material.

**Table 2:** Statistical assessment results for each dataset.

**Dataset**	**Alarm approach**		**Sample approach**	
	**Validated patients** ^+^	**% Validated patients** ^+^	**Validated patients** ^+^	**% Validated patients** ^+^
EPILEPSIAE	5	12.5	39	97.5
CHB-MIT	1	16.7	6	100
AES	-	-	2	28.6
Epilepsy Ecosystem	-	-	3	100

^+^Refer to Section 3.1 for more details.

The discrepancy in results between approaches is evident. Out of the 46 patients studied, only 6 demonstrated performance surpassing the chance level in the alarm-based method. This subset includes five individuals from the EPILEPSIAE dataset (8,902, 32,702, 80,702, 93,402, and 110,602), as well as one from CHB-MIT (chb01). On the other hand, in the sample-based approach, the majority of patients demonstrated performance surpassing the chance level. Among the 56 patients studied, 50 surpassed the surrogate predictor. This difference in values between approaches was already anticipated based on prior findings. The conservative selection of a 0.7 threshold for Firing Power, along with increased rigor in the alarm approach, also influences these suboptimal results. However, this conservative limit precisely ensures that FPR/h values remain within an acceptable range.

Despite comprehensive database comparisons, it is crucial to acknowledge the complexity of this task due to numerous variables and substantial differences in data organization. Even with a method that maintains a high level of rigor, the distinct organization of data and the presence of diverse information introduce complexities in standardizing the process.

### 3.2 Comparison with the state-of-the-art

As shown in Table 3, we chose eight studies to facilitate comparisons and gain insights into the primary distinctions observed when juxtaposed with the current state-of-the-art. This set comprises four studies with the EPILEPSIAE database, three with CHB-MIT data, two with AES, and one with data from the Epilepsy Ecosystem.

**Table 3:** Seizure prediction performance for studies under comparison.

**Database**	**Study**	**No. of Patients**	**SPH (min)**	**SS**	**FPR/h**	**SP**	**AUC**	**Validated Patients^+^**
CHB-MIT	Li et al., 2023	18	1	0.97	0.06	0.87	0.94	-
	Xu et al., 2023	4	5	0.91	0.11	-	0.89	-
	Truong et al., 2018	13	5	0.81	0.16	-	-	92%
	Our Proposed Methodology	6	10	0.28	0.53	0.58	0.52	17%
	Our Validated Patients^+^	1	10	1	0.31	0.46	0.62	-
EPILEPSIAE	Lopes et al., 2023	41	10	0.34	0.90	-	-	51%
	Pinto et al., 2022	93	-	0.16	0.21	-	-	32%
	Pinto et al., 2021	19	10	0.37	0.79	-	-	32%
	Alvarado-Rojas et al., 2014	53	1	0.47	0.94	-	-	13%
	Our Proposed Methodology	40	10	0.13	0.36	0.69	0.56	12.5%
	Our Validated Patients^+^	5	10	0.67	0.24	0.74	0.72	-
AES	Li et al., 2023	4	5	0.93	0.03	0.92	0.97	-
	Truong et al., 2018	7	5	0.75	0.21	-	-	86%
	Our proposed methodology	7	10	0.48	-	0.64	0.56	29%
	Our validated patients^+^	2	10	0.75	-	0.66	0.71	-
Epilepsy ecosystem	Stojanović et al., 2020	3	5	0.69	-	0.79	-	-
	Our Proposed Methodology	3	10	0.75	-	0.37	0.54	100%
	Our validated patients^+^	3	10	0.75	-	0.37	0.54	-

^+^Refer to Section 3.1 for more details.

In analyzing studies using EPILEPSIAE data, it is noteworthy that the sensitivity value achieved by the developed methodology occupies the least favorable position in the table. The only study exhibiting a similar sensitivity value is the one conducted by Pinto et al. (2022). However, it is crucial to note that Pinto's study examined a significantly larger patient population. Additionally, this study employs a simplistic classifier, unlike the approach adopted by Lopes et al. (2023), which incorporates Deep Learning (DL) methods. Therefore, the expectation in the present study was a lower sensitivity value. Nevertheless, the FPR/h value achieved is notably well-positioned, surpassed only by the value obtained by Pinto et al. (2022).

When examining the percentage of statistically validated patients, it is clear that Pinto et al. (2021), Pinto et al. (2022), and Lopes et al. (2023) hold an advantage, presenting a higher value. Alvarado-Rojas et al. (2014) on the other hand, achieved a lower percentage of validated patients. Despite this lower value, it is noteworthy that the approach employed for statistical assessment differed, with utilizing the random predictor.

Regarding the CHB-MIT dataset, all studies demonstrated superior performance in both sensitivity and FPR/h compared to the developed methodology. Moreover, the AUC values obtained by Li et al. (2023) and Xu et al. (2023) were significantly higher. Concerning statistical assessment, only (Truong et al., 2018) executed this stage, achieving an impressive assessment percentage of 92%, exceeding the results obtained in this study. However, it is essential to recognize that the approach employed by Truong et al. (2018) differed, involving the use of the random predictor.

In the context of AES, it is evident that the metrics attained by Truong et al. (2018) and Li et al. (2023) greatly outperformed those attained by the developed algorithm. Significantly, Truong et al. (2018) scored an almost thrice higher statistical assessment rate (86%) than this study's rate (29%).

For the Epilepsy Ecosystem dataset, the scarcity of studies complicates comparative analyses. Our developed algorithm diverges from the prevailing trend seen in previous databases, exhibiting an improved sensitivity value (0.75) in comparison to Stojanović et al. (2020) (0.69). Nevertheless, the specificity registers a considerable decline (0.37 compared to 0.79). It is crucial to note that Stojanović et al. (2020) did not undertake statistical assessment, a critical factor for comparisons, as all participants in our study exhibited performance above chance levels.

Another noteworthy consideration is the selection of the SPH value. Studies opting for a 10-min SPH demonstrated results closely resembling those obtained by our proposed methodology. In contrast, studies employing shorter SPH values, equal to or less than 5 min, showcased improved outcomes. However, it is crucial to highlight that excessively short SPH values may compromise the effectiveness of rescue medication administration, given the time required for medication to exert its therapeutic effects (Bouw et al., 2021).

## 4 Conclusion

This study aimed to develop a methodology for predicting epileptic seizures and facilitating comparisons across four distinct databases. We devised a patient-specific seizure prediction algorithm, following the prevailing pipeline in the literature for EPILEPSIAE data, and adapted it for CHB-MIT, AES, and the Epilepsy Ecosystem datasets.

For the EPILEPSIAE and CHB-MIT datasets, we used alarm triggering due to the availability of temporal seizure data. In contrast, for the AES and Epilepsy Ecosystem datasets without temporal seizure data, we only implemented a sample approach. To maintain methodological consistency and facilitate comprehensive database comparisons, the sample approach was also applied to the EPILEPSIAE and CHB-MIT data.

The evaluation of results leads to a clear conclusion. Dealing with the problem less rigorously, without considering the temporal aspect of seizure occurrence and disregarding long-term interictal data, yields better results. However, this enhanced performance may not translate into a more accurate representation of real-life scenarios; it may even have the opposite effect. Indeed, assumptions crafted to simulate real-life alarm situations result in unfavorable outcomes, as evidenced by the results derived from the EPILEPSIAE and CHB-MIT datasets. Nonetheless, these assumptions remain crucial for addressing the problem and ensuring practical applicability. Thus, achieving impressive results proves inconsequential if they lack realism.

The conclusions drawn from comparing test results with outcomes from other studies that use identical databases align closely. The initial expectation was for slightly weaker results due to the use of a relatively simple pipeline, but the extent of the observed decline was unexpected. Once again, the prevailing belief is that meticulous care and assumptions made to enhance the representation of real-life scenarios led to these low results.

The discrepancies in the CHB-MIT data stand out prominently in all comparisons with other studies. The assumptions employed to simulate real-life conditions consistently yield inferior results, a trend confirmed by observing this pattern even with datasets recognized for their high performance in the majority of available studies, such as the CHB-MIT. Nevertheless, the attainment of positive outcomes loses significance if predicated on unrealistic assumptions that fail to align with the actual experiences of individuals living with epilepsy.

Moreover, the lack of statistical assessment in most of these studies challenges straightforward comparisons. Additionally, several authors opt to mention or accentuate their most favorable results, introducing an element of bias in comparative assessments. For instance, a closer look at the performance metrics obtained with the methodology we developed, considering all patients vs. validated patients, underscores the temptation to present only the most impressive outcomes. However, adopting such a selective approach would compromise the accuracy of representation to reality.

Despite clear conclusions, extracting definitive insights from the obtained results remains challenging. The numerous variables at play make it difficult to pinpoint specific factors contributing to the observed differences in values. Even with a meticulous methodology, the diverse types of data, organizational structures, and accessibility across different databases introduce substantial complexity to the standardization process.

To address these limitations, future efforts should replicate this study using extensive, systematically structured, and fully annotated long-term datasets. This requires acquiring and disseminating additional data in public databases. It is crucial to ensure that this new data is collected in environments mirroring the patient's everyday life. Subsequently, making this data easily accessible to the public, along with essential information for a realistic problem-solving approach, is crucial. Furthermore, the developed methodology should undergo tests with parameter variations, exploring alternative classifiers and standardizing the preprocessing stage to evaluate resulting disparities.

In addition to the limitations above, it is crucial to discuss the real-world applicability of our model, including the realism of preprocessing time. We took considerable care to ensure that our preprocessing phase aligns with real-time constraints. Drawing from a validated approach documented in prior research (Lopes et al., 2021), our methodology was designed to be efficient and applicable within practical timeframes. Furthermore, our deliberate selection of simple classifiers and univariate features aimed to streamline computational demands, enhancing the feasibility of real-time implementation and underscoring the potential impact of our research in the field of seizure prediction (Teixeira et al., 2011).

## Data availability statement

The datasets analyzed for this study are available from the following sources: the EPILEPSIAE dataset at http://www.epilepsiae.eu/, the AES dataset on seizure prediction at https://www.kaggle.com/c/seizure-prediction, the CHB-MIT dataset at https://physionet.org/content/chbmit/1.0.0/, and the Epilepsy Ecosystem dataset at https://www.epilepsyecosystem.org/. Access to the EPILEPSIAE and Epilepsy Ecosystem datasets may be restricted and require permission from the respective data providers.

## Ethics statement

The studies involving humans were approved by the EPILEPSIAE: Approval for the utilization of this data for research purposes was granted by the Ethics Committee of the three hospitals involved in the development of the EPILEPSIAE database (Ethik-Kommission der Albert-Ludwigs-Universität, Freiburg; Comité consultatif sur le traitement de l'information en matiére de recherche dans le domaine de la santé, Hospital Universitário Pitié-Salpětriére; and Ethics Committee of the Centro Hospitalar e Universitário de Coimbra). All studies followed applicable guidelines and regulations, with written informed consent obtained from each patient. CHB-MIT: The data is available for research purposes, and access is open to all, subject to specified terms. AES: Access to the data in this dataset is public, and its usage was permitted after the conclusion of the Kaggle competition. Epilepsy Ecosystem: Accessing and using this data involved completing a form, undergoing security procedures, and consenting to the specified terms of use. The studies were conducted in accordance with the local legislation and institutional requirements. Written informed consent for participation was not required from the participants or the participants' legal guardians/next of kin in accordance with the national legislation and institutional requirements.

## Author contributions

IA: Conceptualization, Investigation, Methodology, Software, Writing – original draft. CT: Conceptualization, Funding acquisition, Project administration, Resources, Supervision, Writing – review & editing. MP: Conceptualization, Data curation, Investigation, Methodology, Validation, Writing – review & editing.
