# Supplementary material for: On the performance of seizure prediction machine learning methods across different databases: the sample and alarm-based perspectives
Source: Front Neurosci. 2024 Jul 15;18:1417748. doi: 10.3389/fnins.2024.1417748 (PMC11284155; doi:10.3389/fnins.2024.1417748)
Supplement: Supplementary file 1 [file Data_Sheet_1.PDF]

Supplementary Material

1 SUPPLEMENTARY DATA

Table S1 provides demographic information for each patient (sex and age) and details about their seizures. The data was obtained from the EPILEPSIAE database, and every patient suffers from Temporal Lobe Epilepsy.

Table S1: Detailed information of the 40 EPILEPSIAE patients.

Patient ID	Age	Sex	Number of seizures (train/test)	Seizure classification	Seizure activity pattern	Vigilance at seizure onset	Recording duration (h)
402	55	f	3	FOIA, FBTC, FOIA	t, t, t	A, A, A	103.81
			2	FBTC, FOIA	t, t	A, A	29.66
8902	67	f	3	UC, FOIA, FOIA	a, b, a	A, A, A	133.91
			2	FOIA, FOIA	m, a	A, A	22.5
11002	41	m	3	UC, FOIA, FOIA	?, s, a	A, R, A	97.16
			1	FOIA	t	A	11.7
16202	46	f	3	UC, FBTC, UC	r, ?, r	A, A, A	201.32
			4	FOIA, FOIA, FOIA, FOIA	r, r, ?, r	A, A, A, A	34.45
21902	47	m	3	UC, FOIA, FOIA	t, t, t	A, A, A	67.08
			1	FOIA	b	R	9.76
23902	36	m	3	FOA, FOA, FOA	t, t, t	A, A, A	70.74
			2	FOA, FOA	d, t	A, A	33.95
26102	65	m	3	FOIA, FOIA, FOIA	m, t, t	A, A, A	60.65
			1	FOIA	t	A	22.58
30802	28	m	3	FOA, FOA, FOA	t ,t ,t	R, A, 2	87.57
			5	FOA, FOA, FOA, FOA, FOA	t, t, t, t, t	A, A, R, 2, 2	61.71
32702	62	f	3	FOIA, FOIA, FOIA	t ,t ,t	A, A, A	117.38
			2	FOIA, FOIA	r, a	A, A	20.49
45402	41	f	3	FOIA, FOIA, FOA	t, t, t	A, A, A	71.98
			1	FOIA	t	A	22.31
46702	15	f	3	FOA, FOIA, FOIA	a, a, t	A, 2, A	47.46
			2	FBTC, FOIA	b, t	2, A	12.6
50802	43	m	3	FOIA, UC, UC	t, t, t	A, 2, 2	165.93
			2	FOIA, FBTC	t, t	2, A	35.6
52302	61	f	3	UC, FOA, UC	?, ?, d	A, A, 1	76.45
			1	UC	t	A	6.85
53402	39	m	3	FOA, FOA, FOA	?, ?, ?	A, 2, A	70.31
			1	FOIA	t	A	13.73
55202	17	f	3	FOIA, FOIA, FOA	t, d, t	A, A, A	47.05
			5	UC, UC, FOA, UC, FOIA	t, t, t, r, r	A, A, A, A, A	65.37
56402	47	m	3	UC, UC, UC	t, ?, ?	A, A, A	184.22
			1	FBTC	a	A	20.25
58602	32	m	3	FOIA, FOIA, FOIA	r, t, t	A, R, A	96.94
			3	FOIA, FOIA, FOIA	r, r, t	A, A, 2	23.34
59102	47	m	3	FOA, FOIA, FOIA	?, t, t	A, A, A	65.83
			2	FOIA, FOA	t, t	A, A	82.22
60002	55	m	3	FOIA, FOIA, FOIA	d, c, t	1, A, A	208.11
			3	UC, FOIA, FOIA	t, d, d	R, R, 1	152.4

Table S1 – *Continued from previous page*

Patient ID	Age	Sex	Number of seizures (train/test)	Seizure classification	Seizure activity pattern	Vigilance at seizure onset	Recording duration (h)
64702	51	m	3	FOA, FBTC, FBTC	?, m, t	A, A, A	75.91
			2	FBTC, FBTC	t, t	A, 2	31.59
75202	13	m	3	FOA, FOA, UC	t, t, t	2, 2, A	100.94
			4	FOA, FOA, FOA, FOA	t, t, ?, t	A, A, A, A	52.63
80702	22	f	3	FOIA, FOIA, UC	b, b, ?	A, A, A	49.4
			3	FOIA, FBTC, FOIA	c, c, c	A, A, A	29.55
85202	54	f	3	FOIA, FOIA, UC	m, c, m	2, A, A	53.49
			2	UC, UC	m, m	A, A	20.42
93402	67	m	3	FBTC, FOIA, FOIA	t, t, t	2, 2, 2	98.0
			2	UC, UC	t, t	2, 2	54.07
93902	50	m	3	FOA, FOIA, FBTC	t, t, d	A, A, 2	370.83
			3	FOIA, FOIA, UC	d, d, d	A, 2, A	20.29
94402	37	f	3	FOA, UC, FOIA	?, d, b	A, A, A	120.23
			4	UC, FOA, UC, FOA	t, ?, b, ?	2, A ,2, A	30.37
95202	50	f	3	FBTC, FOIA, FOIA	b, b, b	2, 2, 2	57.6
			4	FOIA, UC, FOIA, UC	m, b, b, t	2, 2, 2, 2	89.53
96002	58	m	3	FOIA, FOIA, FOIA	t, t, t	A, A, A	48.4
			4	FOIA, UC ,FOIA, FOIA	d ,a ,t ,a	A, A, A, A	82.2
98102	36	m	3	FOA, UC, UC	?, ?, ?	A, A, A	108.61
			2	UC, FBTC	?, ?	A, A	45.68
98202	39	m	3	FOIA, FOIA, FOIA	t, a, t	A, A, A	111.33
			5	FBTC, FOIA, FOIA, FOIA, UC	t, t, t, t, t	A, A, A, A, A	49.88
101702	52	m	3	FOIA, FOIA, FOIA	t, t, t	A, A, A	28.41
			2	FOIA, FOIA	r, r	2, A	23.83
102202	17	m	3	FOA, UC, FOIA	b, ?, t	2, A, 2	57.45
			4	UC, FOA, FOIA, UC	?, t, t, t	A, A, 2, A	51.41
104602	17	f	3	FOIA, FBTC, FBTC	t, a, t	A, 2, 2	87.87
			2	FBTC, UC	t, d	2, 2	15.25
109502	50	m	3	FOIA, FOIA, UC	t, t, t	A, A, A	76.8
			1	UC	t	A	41.94
110602	56	m	3	FOIA, FOIA, FOIA	t, t, t	A, A, A	89.63
			2	FOIA, FOA	t, t	A, A	25.92
112802	52	m	3	UC, FOIA, UC	t, t, t	A, A, A	71.58
			3	FOIA, FOIA, UC	t, t, t	A, A, A	111.5
113902	29	f	3	UC, FOIA, FOIA	t, d, t	A, A, 2	61.98
			3	FOIA, UC, FOIA	t, t, t	A, 2, A	22.73
114702	22	f	3	FOIA, FOIA, UC	t, t, t	A, A, A	68.39
			5	FOIA, FOIA, FOIA, FOIA, FOIA	t, d, t, d, t	A, A, A, A, A	34.04
114902	16	f	3	FOA, FOIA, FOIA	s, b, s	A, A, A	26.55
			4	FBTC, UC, FOIA, FOIA	t, r, a, t	2, A, A, A	50.66
123902	25	f	3	FBTC, FBTC, FOIA	t, t, t	2, 2, R	152.11
			2	FOIA, FOA	t, t	A, A	30.15

Sex: female (f), male (m); Seizure classification: unclassified (UC), Focal Onset Aware (FOA), Focal Onset Impaired (FOIA), Focal to Bilateral Tonic-Clonic (FBTC); Seizure activity pattern: unclear (?), rhythmic sharp waves (s), alpha waves (a), rhythmic delta waves (d), rhythmic theta waves (t), rhythmic beta waves (b), repetitive spiking (r), cessation of interictal activity (c), amplitude depression (m); Vigilance state: awake (A), REM sleep stage (R), Non-REM sleep stage I (1), Non-REM sleep stage II (2).

Tables S2, S3, S4 contain detailed data characteristics for CHB-MIT, AES, and Epilepsy Ecosystem datasets, respectively.

Table S2: Detailed information of the 6 CHB-MIT cases.

Patient	Age	Sex	Number of channels	Number of seizures (train/test)	Recording duration (h)
**chb01**	11	f	23	3	10.59
				1	4.83
**chb06**	1.5	f	23	3	8.19
				3	31.95
**chb10**	3	m	23	3	4.18
				3	111.24
**chb14**	9	f	23	3	8.3
				1	9.32
**chb15**	16	m	32	3	9.75
				5	36.56
**chb24**	-	-	23	3	7.83
				1	5.41

Sex: female (f), male (m).

Table S3: Detailed information of the 7 AES subjects.

Subject	Number of channels	Sampling rate (Hz)	Number of train files	Number of test files	Duration (hours)
**Dog 1**	16	400	468	12	80
**Dog 2**	16	400	476	24	83.3
**Dog 3**	16	400	1392	48	240
**Dog 4**	16	400	742	62	134
**Dog 5**	15	400	426	24	75
**Patient 1**	15	5000	38	12	8.3
**Patient 2**	24	5000	30	12	7

Table S4: Detailed information of the 3 Epilepsy Ecosystem patients.

Patient	Age	Sex	Number of channels	Sampling rate (Hz)	Number of train files	Number of test files	Recording duration (h)
**1**	21	f	16	400	826	62	148.0
**2**	51	f	16	400	2058	297	392.5
**3**	50	f	16	400	2163	206	394.8

Sex: female (f), male (m).

Tables S5, S6, S7, S8 contain the testing results of each patient's metrics for EPILEPSIAE, CHB-MIT, AES, and Epilepsy Ecosystem databases, respectively.

Table S5: Testing results for the EPILEPSIAE database.

Patient	Tested Seizures	FPR/h	SS_{Alarm}	SS_{Sample}	SP_{Sample}	AUC
**402**	2	0.00	0.00	0.34	0.64	0.49
**8902**	2	0.10	1.00	0.96	0.92	0.94
**11002**	1	0.71	0.00	0.50	0.51	0.50
**16202**	4	0.03	0.00	0.07	0.91	0.49
**21902**	1	0.00	0.00	0.01	0.88	0.45
**23902**	2	1.09	0.50	0.68	0.45	0.56
**26102**	1	0.00	0.00	0.26	0.68	0.47
**30802**	5	0.37	0.20	0.68	0.75	0.71
**32702**	2	0.05	0.50	0.50	0.86	0.68
**45402**	1	0.55	0.00	0.51	0.61	0.56
**46702**	2	0.00	0.00	0.41	0.58	0.49
**50802**	2	0.26	0.00	0.23	0.87	0.55
**52302**	1	0.94	0.00	0.53	0.30	0.41
**53402**	1	0.27	0.00	0.74	0.65	0.70
**55202**	5	0.52	0.20	0.66	0.64	0.65
**56402**	1	0.51	0.00	0.42	0.64	0.53
**58602**	3	0.52	0.00	0.49	0.58	0.53
**59102**	2	0.99	0.50	0.76	0.52	0.64
**60002**	3	0.05	0.00	0.19	0.68	0.43
**64702**	2	0.52	0.00	0.48	0.65	0.57
**75202**	4	0.04	0.00	0.18	0.87	0.52
**80702**	3	0.27	0.33	0.58	0.52	0.55
**85202**	2	0.11	0.00	0.11	0.87	0.49
**93402**	2	0.46	1.00	0.85	0.69	0.78
**93902**	3	0.12	0.00	0.32	0.79	0.56
**94402**	4	0.71	0.00	0.40	0.49	0.45
**95202**	4	0.34	0.00	0.40	0.78	0.59
**96002**	4	0.52	0.25	0.25	0.64	0.45
**98102**	2	0.12	0.00	0.50	0.83	0.66
**98202**	5	0.02	0.00	0.33	0.67	0.50
**101702**	2	0.66	0.00	0.46	0.58	0.52
**102202**	4	0.04	0.00	0.21	0.85	0.53
**104602**	2	0.37	0.00	0.51	0.62	0.57
**109502**	1	1.95	0.00	0.36	0.36	0.36
**110602**	2	0.31	0.50	0.61	0.71	0.66
**112802**	3	0.68	0.33	0.53	0.64	0.59
**113902**	3	0.05	0.00	0.36	0.68	0.52
**114702**	5	0.00	0.00	0.20	0.80	0.50
**114902**	4	0.00	0.00	0.28	0.92	0.60
**123902**	2	0.00	0.00	0.00	0.99	0.50
**Overall**	1.00±2.06	0.36±0.40	0.13±0.26	0.42±0.22	0.69±0.16	0.56±0.11

SS_{Alarm} stands for Alarm Sensitivity, SS_{Sample} for Sample Sensitivity, SP_{Sample} for Sample Specificity, and AUC for Area Under the Curve.

Table S6: Testing results for the CHB-MIT database.

Patient	Tested Seizures	FPR/h	SS_{Alarm}	SS_{Sample}	SP_{Sample}	AUC
**chb01**	1	0.31	1.00	0.77	0.46	0.62
**chb06**	3	0.89	0.33	0.91	0.46	0.68
**chb10**	3	0.38	0.33	0.49	0.57	0.53
**chb14**	1	0.52	0.00	0.19	0.51	0.35
**chb15**	5	0.10	0.00	0.00	0.91	0.46
**chb24**	1	0.95	0.00	0.35	0.56	0.46
**Overall**	2.33±1.63	0.53±0.34	0.28±0.39	0.45±0.34	0.58±0.17	0.52±0.12

SS_{Alarm} stands for Alarm Sensitivity, SS_{Sample} for Sample Sensitivity, SP_{Sample} for Sample Specificity, and AUC for Area Under the Curve.

Table S7: Testing results for the AES database.

Patient	Tested files (preictal\interictal)	SS_{Sample}	SP_{Sample}	AUC
**Dog 1**	468 (6\462)	0.50	0.54	0.52
**Dog 2**	482 (12\470)	0.75	0.79	0.77
**Dog 3**	1416 (24\1392)	0.35	0.76	0.56
**Dog 4**	769 (31\738)	0.41	0.67	0.54
**Dog 5**	444 (12\432)	0.76	0.54	0.65
**Patient 1**	44 (6\38)	0.40	0.50	0.45
**Patient 2**	36 (6\30)	0.16	0.69	0.42
**Overall**	-	0.48±0.22	0.64±0.12	0.56±0.12

SS_{Sample} stands for Sample Sensitivity, SP_{Sample} for Sample Specificity, and AUC for Area Under the Curve.

Table S8: Testing results for the Epilepsy Ecosystem database.

Patient	Tested files (preictal\interictal)	SS_{Sample}	SP_{Sample}	AUC
**Patient 1**	72 (23\49)	0.80	0.43	0.61
**Patient 2**	305 (18\287)	0.71	0.45	0.58
**Patient 3**	207 (18\189)	0.73	0.24	0.42
**Overall**	-	0.75±0.05	0.37±0.12	0.54±0.10

SS_{Sample} stands for Sample Sensitivity, SP_{Sample} for Sample Specificity, and AUC for Area Under the Curve.

Table S9 contains the statistical validation results of each patient's metrics for EPILEPSIAE, CHB-MIT, AES, and Epilepsy Ecosystem databases.

Table S9: Statistical validation results for all databases.

Patient	SS_{Alarm}	SS_{Alarm} Surrogate	p-value Alarm	SS_{Sample}	SS_{Sample} Surrogate	p-value Sample	Above chance (Alarm)	Above chance (Sample)
**EPILEPSIAE**								
**402**	0.00	0.00	1.00	0.34	0.00	0.00		•
**8902**	1.00	0.13	2.97×10^{-15}	0.96	0.00	1.18×10^{-86}	•	•
**11002**	0.00	0.17	1.00	0.50	0.00	1.77×10^{-73}		•
**16202**	0.00	0.03	1.00	0.07	0.00	0.00		•
**21902**	0.00	0.00	1.00	0.01	0.00	0.00		•
**23902**	0.50	0.42	0.86	0.68	0.00	1.96×10^{-84}		•
**26102**	0.00	0.00	1.00	0.26	0.00	0.00		•
**30802**	0.20	0.39	1.00	0.68	0.00	2.31×10^{-86}		•
**32702**	0.50	0.03	2.01×10^{-10}	0.50	0.00	2.64×10^{-81}	•	•
**45402**	0.00	0.27	1.00	0.51	0.00	2.82×10^{-73}		•
**46702**	0.00	0.00	1.00	0.41	0.00	0.00		•
**50802**	0.00	0.07	1.00	0.23	0.00	2.95×10^{-68}		•
**52302**	0.00	0.27	1.00	0.53	0.00	6.06×10^{-82}		•
**53402**	0.00	0.10	1.00	0.74	0.00	1.10×10^{-95}		•
**55202**	0.20	0.32	1.00	0.66	0.00	1.61×10^{-84}		•
**56402**	0.00	0.20	1.00	0.42	0.00	2.03×10^{-69}		•
**58602**	0.00	0.12	1.00	0.49	0.00	2.61×10^{-73}		•
**59102**	0.50	0.42	0.36	0.76	0.00	9.01×10^{-83}		•
**60002**	0.00	0.04	1.00	0.19	0.00	3.43×10^{-74}		•
**64702**	0.00	0.23	1.00	0.48	0.00	1.45×10^{-81}		•
**75202**	0.00	0.01	1.00	0.18	0.00	1.63×10^{-78}		•
**80702**	0.33	0.18	1.82×10^{-8}	0.58	0.00	9.02×10^{-86}	•	•
**85202**	0.00	0.05	1.00	0.11	0.00	2.95×10^{-64}		•
**93402**	1.00	0.23	1.37×10^{-9}	0.86	0.00	6.25×10^{-79}	•	•
**93902**	0.00	0.03	1.00	0.32	0.00	4.67×10^{-85}		•
**94402**	0.00	0.24	1.00	0.40	0.00	1.76×10^{-77}		•
**95202**	0.00	0.12	1.00	0.40	0.00	4.28×10^{-77}		•
**96002**	0.25	0.18	0.14	0.25	0.00	6.16×10^{-68}		•
**98102**	0.00	0.07	1.00	0.50	0.00	2.55×10^{-82}		•
**98202**	0.00	0.03	1.00	0.33	0.00	1.15×10^{-78}		•
**101702**	0.00	0.28	1.00	0.46	0.00	3.94×10^{-81}		•
**102202**	0.00	0.01	1.00	0.21	0.00	2.13×10^{-87}		•
**104602**	0.00	0.08	1.00	0.51	0.00	1.96×10^{-82}		•
**109502**	0.00	0.43	1.00	0.36	0.00	8.57×10^{-71}		•

Table S9 – *Continued from previous page*

Patient	SS_{Alarm}	SS_{Alarm} Surrogate	p-value Alarm	SS_{Sample}	SS_{Sample} Surrogate	p-value Sample	Above chance (Alarm)	Above chance (Sample)
**110602**	0.50	0.15	3.66×10^{-7}	0.61	0.00	3.56×10^{-84}	•	•
**112802**	0.33	0.26	0.08	0.53	0.00	7.79×10^{-69}		•
**113902**	0.00	0.06	1.00	0.36	0.00	0.00		•
**114702**	0.00	0.00	1.00	0.20	0.00	0.00		•
**114902**	0.00	0.00	1.00	0.29	0.00	0.00		•
**123902**	0.00	0.00	1.00	0.00	0.00	1.00		
**CHB-MIT**								
**chb01**	1.00	0.07	5.58×10^{-10}	0.77	0.00	1.69×10^{-86}	•	•
**chb06**	0.33	0.40	1.00	0.91	0.00	1.63×10^{-90}		•
**chb10**	0.33	0.44	1.00	0.49	0.00	8.79×10^{-48}		•
**chb14**	0.00	0.13	1.00	0.19	0.00	1.01×10^{-69}		•
**chb15**	0.00	0.01	1.00	0.004	0.000	1.54×10^{-25}		•
**chb24**	0.00	0.20	1.00	0.35	0.00	6.31×10^{-73}		•
**AES**								
**Dog 1**	-	-	-	0.5012	0.5503	1.00	-	
**Dog 2**	-	-	-	0.7481	0.4811	8.02×10^{-41}	-	•
**Dog 3**	-	-	-	0.3493	0.5140	1.00	-	
**Dog 4**	-	-	-	0.4091	0.5194	1.00	-	
**Dog 5**	-	-	-	0.7596	0.3390	3.34×10^{-45}	-	•
**Patient 1**	-	-	-	0.4036	0.5994	1.00	-	
**Patient 2**	-	-	-	0.1619	0.2675	1.00	-	
**Epilepsy Ecosystem**								
**Patient 1**	-	-	-	0.7985	0.4301	2.94×10^{-47}	-	•
**Patient 2**	-	-	-	0.7144	0.3465	2.16×10^{-49}	-	•
**Patient 3**	-	-	-	0.7315	0.4216	1.67×10^{-45}	-	•

SS_{Sample} stands for Sample Sensitivity, and SP_{Sample} for Sample Specificity.
